# Prognostic significance of chemotherapy-induced necrosis in osteosarcoma patients receiving pasteurized autografts

**DOI:** 10.1371/journal.pone.0172155

**Published:** 2017-02-14

**Authors:** Min Wook Joo, Yong Koo Kang, Chang-Young Yoo, Sung Ho Cha, Yang-Guk Chung

**Affiliations:** 1 Department of Orthopaedic Surgery, St. Vincent's Hospital, College of Medicine, The Catholic University of Korea, Seoul, Republic of Korea; 2 Department of Hospital Pathology, St. Vincent's Hospital, College of Medicine, The Catholic University of Korea, Seoul, Republic of Korea; 3 Department of Orthopaedic Surgery, Seoul St. Mary's Hospital, College of Medicine, The Catholic University of Korea, Seoul, Republic of Korea; Universidad de Navarra, SPAIN

## Abstract

**Background:**

Among various reconstruction methods after wide excision for osteosarcoma, pasteurized autograft is often preferred. While the whole area of the tumor can be assessed for chemotherapy-induced necrosis, one of the important prognostic factors, in other reconstructive techniques, only a portion removed from a wide-resection specimen is available when using pasteurized autograft method. The assessment, therefore, may be unreliable. We analyzed the prognostic significance of the chemotherapy-induced necrosis in osteosarcoma patients who underwent reconstruction with pasteurized autografts.

**Patients and methods:**

We reviewed the records of osteosarcoma patients who underwent treatment in our institution from 1998 to 2013. Cases of reconstruction with pasteurized autografts were defined as the patient group, and the same number of patients who underwent other reconstruction methods served as controls. Chemotherapy-induced necrosis was evaluated for removed extra-osseous and curetted intramedullary tumor tissues.

**Results:**

A total of 22 patients were identified; the median age was 15.5 years, and there were 12 males. The most common tumor location was the distal femur. The most common histological subtype was osteoblastic. Median size was 8.1 cm. Disease status was stage IIB in 13 patients and IIA in 9. Median follow-up was 76 months. No differences between the patient and control groups were observed in potential prognostic factors, overall survival, metastasis-free survival, or recurrence-free survival. Univariate analyses demonstrated that histological response was a significant prognostic factor for metastasis-free survival and also significant for recurrence-free survival.

**Conclusion:**

Chemotherapy-induced necrosis grading, using only available tumor tissues, could be a prognostic factor for osteosarcoma patients receiving pasteurized autografts for reconstructive surgery.

## Introduction

Before the introduction of chemotherapy, nearly 90% of patients with osteosarcoma developed distant metastases, and died of their disease despite the standard treatment at the time, limb amputation [1,2]. Even though multidisciplinary treatment with pre- and post-operative chemotherapy and wide resection has improved clinical outcomes since the late twentieth century, the 5-year overall survival (OS) rate remains around 60%, and local recurrence and metastasis are not infrequent after completion of initial treatment [3–5]. The 5-year OS rate for those with metastatic disease, less than 30%, has scarcely changed over the past three decades [6,7]. While the pathological mechanisms are not still well understood, neo-vascularization, invasion, anoikis resistance, chemotherapy resistance, and evasion of the immune response are considered to be factors contributing to progression and metastasis in osteosarcoma [8]. To improve oncologic outcomes, definite prognostic factors should be found in order to identify high-risk patients earlier and more efficiently, and prevent metastasis. The published literature suggests that age, tumor volume and site, grade of chemotherapy-induced necrosis, surgical margin, alkaline phosphatase, serum lactate dehydrogenase, and c-reactive protein are important prognostic factors [9–13].

In limb salvage procedures for osteosarcoma in the extremities, reconstruction of skeletal defects after wide excision is essential; therefore, various surgical procedures have been used [14,15]. While tumor-prosthesis is currently the most common reconstruction method for massive skeletal defects, processed allograft, autograft, allograft-prosthesis composite, and autograft-prosthesis composite surgery have also been performed [15–21]. Among different sorts of autografts, including iliac bone, vascularized fibula, and recycled bone grafts, the latter needs to be extra-corporeally processed to devitalize tumor cells shortly before re-implantation of resected bone. There are a variety of pre-treatment methods, such as pasteurization [19], autoclaving [21], irradiation [18], and freeze-and-thawing using liquid nitrogen [20]. Recycled autografts are preferred as they reduce immune response and disease transmission, and improve economic efficiency, accessibility, and anatomical conformation [18–21]. Because pasteurized autografts preserve bone-inductive activity and effectively eliminate viable tumor cells [19], they are mainly used in our institution.

Skeletal reconstruction using recycled autografts including pasteurized autografts, however, entails a critical obstacle. The chemotherapeutic response is usually assessed for cellularity and tumor necrosis in an entire resected bone. While the whole area of the tumor can be evaluated when other reconstruction techniques are used, only a portion of the tumor removed from a wide-resection specimen is available. Therefore, the assessment could be insufficient and unreliable. As mentioned above, the grade of chemotherapy-induced necrosis offers important prognostic information required to determine a treatment plan [2].

To the best of our knowledge, the prognostic significance of the histologic response to neoadjuvant chemotherapy in osteosarcoma patients who underwent reconstruction with pasteurized autografts has not yet been analyzed. We carried out an analysis using data from our medical center.

## Patients and methods

We retrospectively reviewed the medical records of osteosarcoma patients hospitalized in our medical center from October 1998 to February 2013. Patients without sufficient information, with tumors in other sites than the long bone, with secondary osteosarcomas, with initial metastasis, with low-grade osteosarcomas, with inadequate surgical margins, who were not treated by preoperative chemotherapy, who did not complete preoperative chemotherapy, who had definite surgeries at other institutions, who did not undergo curative operations, who were managed by amputation instead of limb salvage surgery, or who did not require reconstruction were excluded from the analysis. Cases of reconstruction with a pasteurized autograft for a skeletal defect following tumor resection were defined as the patient group, and the same number of patients who underwent other types of reconstruction surgery were selected as the control group. Because this study was a retrospective chart review and minimal risk study, and any personally identifiable information was not collected, we did not receive any informed consents from participants. This study was also approved by the Catholic University of Korea St. Vincent’s Hospital Institutional Review Board.

Information on age, gender, symptom duration before diagnosis, location, lesion size, histological subtype, laboratory results, secondary osteosarcoma, American Joint Committee on Cancer (AJCC) stage, metastasis at time of the initial diagnosis, surgery, surgical margins, radiotherapy, chemotherapy, histological response to neoadjuvant chemotherapy, local recurrence, distant metastasis, oncologic results, and follow-up period was identified. Lesion size was defined as the length of the main axis. Definitive surgery was defined as surgery that was intended to achieve a curative margin. Metastasis-free survival (MFS) and Recurrence-free survival (RFS) were defined as the time from the initial diagnosis to date of the first detection of distant metastasis and from date of definitive surgery to date of the first recognition of local recurrence. Follow-up was defined as the period from diagnosis to death or the last follow-up visit.

Patients received three cycles of neoadjuvant chemotherapy using a combination of high-dose methotrexate, adriamycin, cisplatin, and ifosfamide. Each cycle took about five weeks, which was modified for each patient based on their general condition, previous evaluation of chemotherapy-induced toxicity, and renal and liver function. Radiological assessment on the effects of chemotherapy was performed after each cycle of chemotherapy. Additional courses of chemotherapy were not administered to patients who showed disease progression; those patients underwent surgery immediately. After definitive surgery, patients received six additional cycles of chemotherapy, also using high-dose methotrexate, adriamycin, cisplatin, and ifosfamide.

**Fig 1:**
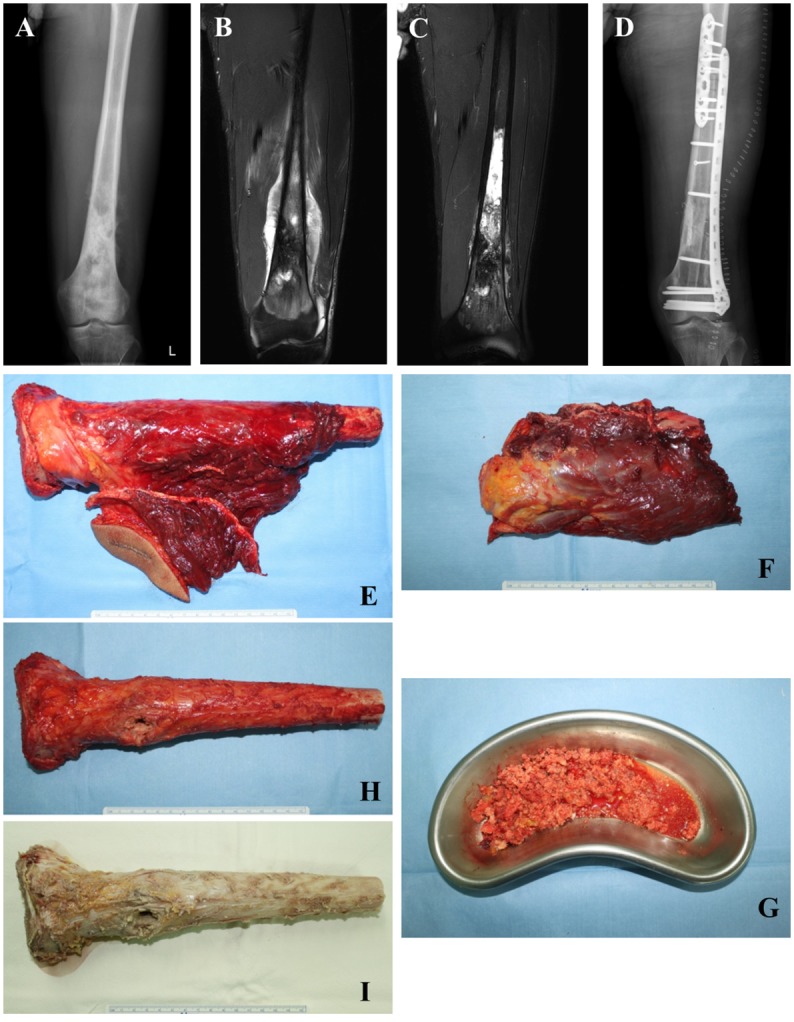
A 21-year-old man with osteosarcoma of the left femur. (A) An anteroposterior radiograph showing an osteoblastic lesion with a periosteal reaction mainly in the metaphysis. (B) An initial T2-weighted coronal MRI scan image showing an ill-defined signal change in the medullar region and soft tissue mass formation in the distal meta-diaphysis of the femur, suggesting a malignant bone tumor. (C) A T2-weighted coronal MRI scan image taken after preoperative chemotherapy showing increased intra-tumoral ossification and decreased extraosseous tumor extension with better-defined margins compared to the initial image. (D) A postoperative anteroposterior radiograph showing an excellent skeletal fit of the pasteurized autograft and augmentation with the in-lay vascularized fibula graft. (E) A wide-excision specimen including a biopsy tract. (F) The removed extra-osseous part and (G) curetted intramedullary tumor tissues from the wide-resection specimen were used for evaluation of necrosis rate. (H) The remaining cortical autograft after removal of extra-osseous and intramedullary tumor tissues. (I) Autograft after pasteurization.

After tumor resection with an adequate margin, soft tissues inclusive of extra-osseous tumors were removed from the wide-excision specimen, and the intramedullary portion of the tumor was also curetted. Removed tissues were used for histological evaluation. The remaining portion of the wide-excision specimen was thoroughly irrigated by saline solution and enclosed in a container filled with previously boiled sterile distilled water at 65°C, and the container was placed into a pasteurization machine. Pasteurization was performed at 65°C for 30 minutes, then the recycled autograft was irrigated again. The skeletal defect of the long bone after wide excision surgery was reconstructed using the pasteurized autograft with additional instruments such as prostheses, intramedullary nails, or plates. A vascularized fibular graft was sometimes added for relatively long segment reconstructions (Fig 1).

Histological responses to preoperative chemotherapy were evaluated for removed extra-osseous and curetted intramedullary tumor tissues. All the masses including the largest cross-section were observed to assess the response to chemotherapy. The response was classified based on percentage of tumor necrosis, as follows: good responder, ≥90%, or poor responder, <90% [22].

Cases in the control group were matched to cases in the patient group based on age, gender, and tumor location and size. The differences between the patient and control groups were assessed with the Mann-Whitney U test for age and size, by Fisher’s exact test for AJCC stage, and by the Chi-square test for histological response. Prognostic differences between the two groups were compared using the log-rank test. The prognostic significance of age (<40 or ≥40 years) [12], gender, lesion size, and histological response to preoperative chemotherapy were analyzed in the patient group. OS, MFS, and RFS were calculated using Kaplan-Meier survival curves. The impact of prognostic factors was assessed using the log-rank test in univariate analysis. A p-value < 0.05 was considered significant. Statistical analysis was done with SPSS 18.0 for Windows (SPSS Corporation, Chicago, IL).

## Results

The clinical characteristics of cases in the patient group are listed in Table 1. A total of 22 patients were identified. There were 12 males and 10 females with a median age of 15.5 years (range, 8–50 years) at the time of initial diagnosis. Median symptom duration before diagnosis was 2 months (range, 0.3–10 months). The most common location was the distal femur (13 cases, 59.1%). The most common histological subtype was osteoblastic osteosarcoma (16 cases, 72.7%). Median tumor size was 8.1 cm (range, 6.4–26.7 cm). Disease status was stage IIB in 13 patients (59.1%) and IIA in 9 (40.9%). Median follow-up period was 76 months (range 15–251 months).

**Table 1:** Characteristics of patients.

No.	Sex	Age (y)	Symptom duration (Mo)	Tumor location	Main length (cm)	Histologic subtype	AJCC stage	Histological response (%)	Local recurrence (Mo)	Distant Metastasis (Mo)	Follow-up (Mo)	Oncologic outcome
1	M	21	4	DF	9.5	PO	IIB	>95			251	CDF
2	F	10	2	DF	8.1	OB	IIB	50	17	64	66	AWD
3	F	12	0.4	DF	11.7	OB	IIB	100		55	74	DOD
4	F	15	1	PH	6.6	OB	IIA	>90			156	CDF
5	M	14	3	DF	7.1	OB	IIA	100			44	CDF
6	M	16	2	DT	6.8	OB	IIA	>90			97	CDF
7	M	18	9	PH	9.3	CB	IIB	50		46	72	DOD
8	F	11	7	PH	8.2	OB	IIB	85			98	CDF
9	M	11	0.3	DF	26.3	OB	IIB	<50			15	CDF
10	M	19	3	DF	9.3	PO	IIB	95			187	CDF
11	F	11	3	DF	8.2	OB	IIB	<50	13	58	63	DOD
12	F	13	0.4	DF	12.0	CO	IIB	100		44	75	DOD
13	F	14	0.5	PH	6.4	OB	IIA	>90			161	CDF
14	M	14	2	DF	6.8	OB	IIA	100			55	CDF
15	M	17	3	DT	6.5	OB	IIA	100			102	CDF
16	M	21	10	PH	9.4	OB	IIB	<80		52	77	DOD
17	F	9	6	PH	8.1	OB	IIB	85			95	CDF
18	M	10	0.8	DF	26.7	CB	IIB	<50			41	CDF
19	M	14	2	PT	7.8	OB	IIA	100			60	CDF
20	M	17	1	DF	8.2	OB	IIB	>90			50	CDF
21	F	40	1	DF	7.9	OB	IIA	95			139	CDF
22	F	54	2	DF	7.2	FB	IIA	>90			132	CDF

*DF* distal femur, *PH* proximal humerus, *DT* distal tibia, *PT* proximal tibia, *PO* periosteal, *OB* osteoblastic, *CB* chondroblastic, *FB* fibroblastic, *CDF* continuous disease free, *AWD* alive with disease, *DOD* died of disease

A comparison between the patient and control groups is shown in Table 2. No differences in potential prognostic factors, 5- year OS, MFS, or RFS were observed, which suggested that the control group was sufficiently similar to the patient group.

**Table 2:** Comparison between patient and control groups.

		Patient group	Control group	*p* value
Age (median)		14.5 (9–54)	15.5 (8–50)	0.311
Gender (Male/Female)		12/10	12/10	
Site	DF	13	13	
	PH	6	6	
	DT	2	2	
	PT	1	1	
Size (cm, median)		8.1 (6.4–26.7)	9.3 (6.6–29.3)	0.074
AJCC stage (IIA/IIB)		9/13	5/17	0.332
Histological response (Good/Poor)		16/6	15/7	1.000
5-year OS (%)		76.7	89.7	0.704
5-year MFS (%)		72.9	57.6	0.165
5-year RFS (%)		90.7	90.9	0.961

*DF* distal femur, *PH* proximal humerus, *DT* distal tibia, *PT* proximal tibia, *OS* overall survival, *MFS* metastasis-free survival, *RFS* recurrence-free survival

**Fig 2:**
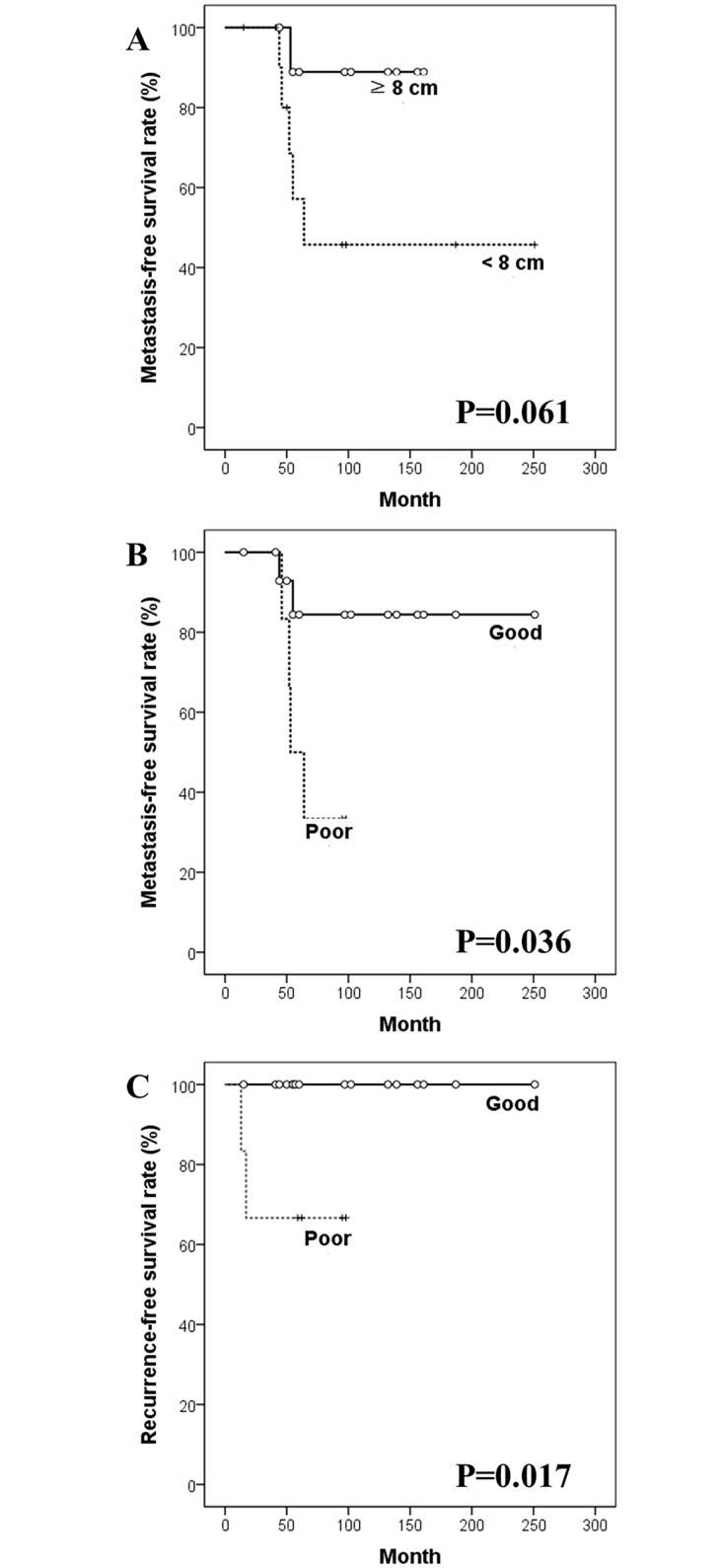
Kaplan-Meier survival curves in univariate analyses. Kaplan-Meier curves for metastasis-free survival according to (A) tumor size, and (B) histological response to pre-operative chemotherapy, and for (C) recurrence-free survival according to histological response.

Table 3 shows the prognostic factors for the patient group. Univariate analyses demonstrated that histological response was a significant prognostic factor for 5-year MFS (p = 0.036) and the difference in MFS according to tumor size approached statistical significance (p = 0.061). In addition, histological response was a significant factor for RFS (p = 0.017) (Fig 2). Prognostic factor analyses for the control group showed that the difference in MFS depending on the grade of chemotherapy-induced necrosis was almost significant (p = 0.071), which additionally implied that the group was analogous to the patient group to be set as a control. The cases in the control group were already matched based on tumor size, and the difference in MFS according to the main length of lesion was also close to significance as well as in the patient group (p = 0.061). More details of prognostic factor analyses on the control group were not shown.

**Table 3:** Prognostic factor analyses.

Factors		*N*	5-year OS (%)	*p* value	5-year MFS (%)	*p* value	5-year RFS (%)	*p* value
Age	< 40 years	20	73.7	0.375	69.6	0.337	89.7	0.646
	≥ 40 years	2	100		100		100	
Gender	Male	12	85.7	0.811	76.2	0.603	100	0.120
	Female	10	70.0		70.0		80.0	
Size	< 8 cm	10	87.5	0.189	88.9	0.061	90.0	0.892
	≥ 8 cm	12	66.7		57.1		90.9	
Histological response	Good	16	82.5	0.233	84.4	0.036*	100	0.017*
	Poor	6	66.7		50.0		66.7	

*OS* overall survival, *MFS* metastasis-free survival, *RFS* recurrence-free survival

*Statistically significant

Local recurrence occurred in 2 patients who underwent reconstruction with recycled autografts. However, magnetic resonance imaging examinations demonstrated that the recurrences were in surrounding soft tissues away from the autografts, and no tumoral invasion into the autografts was observed. In the control group, the same number of patients developed local relapses.

## Discussion

Although allografts are a more commonly used reconstruction tool for the correction of massive skeletal defects after osteosarcoma resection except for tumor-prostheses, recycled autografts are also a useful option [2], In some countries, especially Asian regions, patients may refuse allograft implantation for social and religious reasons [23]. In situations where a bone bank is not available, recycled autografts are readily available and do not require special devices and facilities; therefore, they are economically efficient and easily accessible [24,25]. They do not cause immunological reactions or disease transmission, either [23]. A relatively simple reconstruction procedure can be expected because of the coincidence of the shape of the massive skeletal defect and the reconstructive material used [23].

Pasteurized autograft implantation for mandibular reconstruction after malignant tumor resection was first reported in 1991 [26], and the technique was introduced in the orthopaedic literature in Japanese first in 1993 [27]. Pasteurization of autogenous bone was performed with moderate heat treatment for tens of minutes [28]. The process is relatively simple because only two factors, temperature and time, have to be controlled. It was reported that tumor cells are completely destroyed by treatment at 70°C for 10 min [29], which preserves bone-inductive activity [30]. As heat treatment above 100°C destroys bone-inductive features [30,31], autoclaved autografts cannot produce new bone formation, therefore, is difficult for them to connect with the host bone [32]. A previous experimental animal study demonstrated that the temperature at the center of the cortex reached the target within 2.5 minutes after initiation of pasteurization [31]. Thus, some groups recommended an additional 5-minute procedure for clinical use [25,31]. Our medical center traditionally stuck to treatment at 65°C for 30 minutes. Despite several advantages, reconstruction with pasteurized autografts has been associated with bone resorption, fracture, and non-union [24]. Augmentation with vascularized fibular grafts in cases with larger segmental reconstruction has been reported to have favorable outcomes [33].

Before pasteurization, the wide-resection specimens are first cleared of soft, gross, extra-osseous and intramedullary tumor tissues [25], which are used to assess the grade of chemotherapy-induced necrosis in the same manner as in other recycling processes. If tumor-prostheses or allografts are used for reconstruction of skeletal defect, total histological mapping of tumor destruction is possible to identify the predominant sites of viable and nonviable tumor cells after neoadjuvant chemotherapy [34]. If recycled autografts are re-implanted, the analysis of the entire tumor and assessment by such standard methods are impossible, which can be a problem for pathologists. As a result, the grade of tumor necrosis may not reflect the prognosis. The first study on this issue was performed for patients with high-grade extremity osteosarcoma who underwent reconstruction with autografts frozen and thawed in liquid nitrogen. Multivariate analysis revealed that poor necrosis grade was a significant prognostic factor for overall survival in that study [2]. To the best of our knowledge, this study is the first on the prognostic significance of the tumor necrosis rate induced by neoadjuvant chemotherapy in osteosarcoma patients receiving pasteurized autografts. Histological response to chemotherapy was also demonstrated to be a prognostic factor for RFS in this study.

There are several limitations to the current study. First, the statistical power was limited by the small number of patients because the incidence of osteosarcoma (2–3/million/year) is extremely low [35], pasteurized autografts are only one option among various reconstruction methods [15–21], and many cases were excluded given the exclusion criteria. Thus, we additionally compared potential prognostic factors and survival rates of patient and control groups. Secondly, other known potential prognostic factors were not assessed together. Although we identified laboratory results such as erythrocyte sediment rate, C-reactive protein, lactate dehydrogenase, and alkaline phosphatase, we did not analyze those factors because they too frequently altered depending on patient condition.

## Conclusions

Chemotherapy-induced necrosis grading, using only available tumor tissues, could be a prognostic factor for osteosarcoma patients receiving pasteurized autografts for reconstructive surgery. Prospective studies with more patients will be necessary to reach a definitive conclusion on the predictive value of tumor necrosis state in patients receiving pasteurized tumor-bearing autografts.
